# Emergency escape surgery for a gastro-bronchial fistula with respiratory failure that developed after esophagectomy

**DOI:** 10.1007/s00595-013-0821-0

**Published:** 2014-01-22

**Authors:** Yuta Ibuki, Yoichi Hamai, Jun Hihara, Junya Taomoto, Ichiko Kishimoto, Yoshihiro Miyata, Morihito Okada

**Affiliations:** Department of Surgical Oncology, Research Institute for Radiation Biology and Medicine, Hiroshima University, 1-2-3 Kasumi, Minami-Ku, Hiroshima, 734-8551 Japan

**Keywords:** Esophageal cancer, Gastric tube ulcer, Two-stage operation

## Abstract

A gastro-bronchial fistula (GBF) is a rare complication after esophageal reconstruction using a gastric tube, but it can cause severe pneumonia, and the surgical procedure is challenging. We herein describe a patient who was successfully managed using a two-stage operation for a GBF. Because the patient had life-threatening pneumonia and respiratory failure caused by the GBF, we first transected the duodenum, established a cervical esophagostomy and gastrostomy and placed a decompression catheter in the gastric tube without a thoracotomy. The patient recovered from pneumonia after the resolution of the salivary inflow and digestive juice reflux into the lungs through the GBF. Two months later, an esophageal bypass was achieved by reconstructing the esophagus using a long segment of pedicled jejunum. The patient was discharged 38 days thereafter. Appropriate treatment for GBF should be tailored to individual patients based on their current status and disease severity.

## Introduction

Gastro-bronchial fistulae (GBFs) occasionally arise after esophagectomy [1–3]. Even severe GBFs usually require surgical management, which generally requires thoracotomy and one-lung ventilation. However, such procedures might be too invasive for patients with severe respiratory failure due to the GBF. We previously reported that esophageal bypass surgery without thoracotomy, followed by chemoradiotherapy (CRT), could be a treatment strategy for patients with esophageal cancer with airway infiltration [4].

We herein describe a patient with severe pneumonia and a GBF that developed after esophagectomy, who was successfully managed by a two-stage operation. The first life-saving procedure aimed to improve the pneumonia, and the second was performed to restore esophageal continuity using an esophageal bypass.

## Case report

A 57-year-old male with advanced upper and middle thoracic esophageal cancer underwent a subtotal esophagectomy via the transthoracic approach and a reconstruction via a gastric tube through the posterior mediastinal route at our hospital. The pathological examination revealed poorly differentiated squamous cell carcinoma that had invaded the adventitia and involved the lower thoracic paraesophageal lymph node. Therefore, the pathological diagnosis of the tumor was T3N1M0 Stage III according to the TNM classification of the International Union for Cancer classification [5]. Because the cancer involved tissue near the surgical margin of the cervical esophagus, the patient underwent postoperative CRT.

**Fig. 1:**
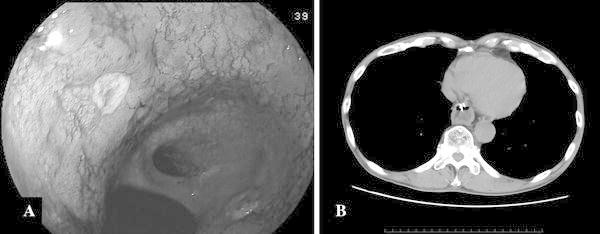
Gastrointestinal fiberscopy and CT images of the gastric tube cancer. **a** The gastric tube cancer was located on the anterior wall of the antrum. **b** The gastric tube cancer was located behind the* left* atrium on computed tomography, and a clip was placed for radiotherapy

Five years later, gastrointestinal fiberscopy and endoscopic ultrasound revealed intra-submucosal gastric tube cancer, which was located on the anterior wall of the antrum (Fig. 1). The pathological diagnosis of biopsy specimens was signet ring cell carcinoma. Computed tomography (CT) did not show either enlarged lymph nodes or distant metastasis.

The gastric tube cancer was considered a contraindication for endoscopic resection because of the histological type and the fact that it had invaded the deep submucosal layer. Moreover, we considered that surgically removing the gastric tube via the posterior mediastinal route would be extremely difficult, and the patient also preferred to avoid this procedure. Thus, we treated the gastric tube cancer by CRT using docetaxel and S-1 with a concurrent total dose of 60 Gy of radiation. A clinical study performed at our institute indicated that the docetaxel/S-1 combination had the potential to improve the survival in patients with advanced or recurrent gastric cancer [6], so we select this regimen for our patients with gastric tube cancer.

This strategy considerably reduced the size of the cancer, and resulted in a complete response. The patient then underwent maintenance chemotherapy with S-1 for 1 year. The patient was monitored biannually by gastrointestinal fiberscopy and CT, and recurrent foci remained undetectable over the follow-up period. However, several chronic ulcers and ulcer scars were sometimes evident in the gastric tube, and the patient continued to take a proton pump inhibitor.

**Fig. 2:**
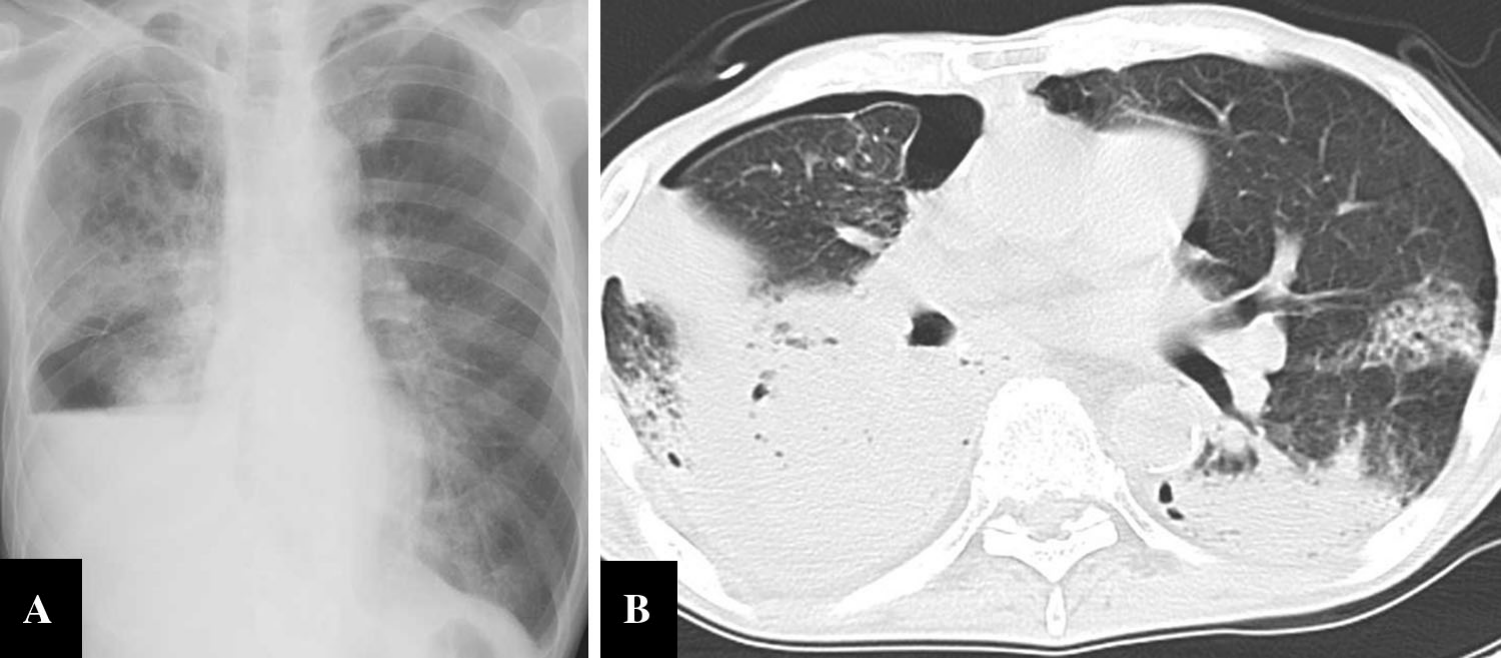
Chest X-ray and CT images upon admission. **a** Lobar pneumonia of the* right* lung, atelectasis of the *right* inferior lobe and *right* pneumothorax. **b** Bilateral atelectasis and pneumonia, and* right* pneumothorax

Three years after the CRT for gastric tube cancer, he visited our hospital on an emergency basis with severe dyspnea and fatigue. A blood gas analysis on admission revealed severe respiratory failure with a PaO_2_ of 65.9 mmHg and a PaCO_2_ of 34.5 mmHg (O_2_, 5 L/min). The findings of chest X-rays and CT images revealed severe pneumonia, atelectasis and pneumothorax (Fig. 2). He was transported to the intensive care unit, where he was intratracheally intubated and placed on a ventilator with 60 % oxygen inhalation.

**Fig. 3:**
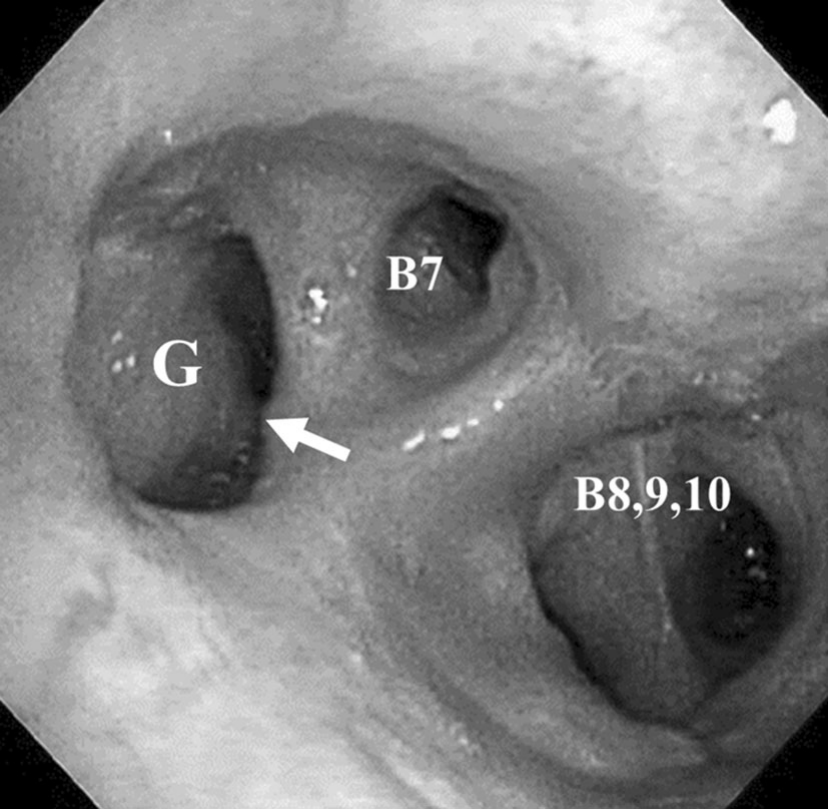
The bronchoscopic image showing a fistula between the base of the *right* inferior lobe bronchus and gastric tube. The diameter of the GBF was about 1 cm. *Arrow* GBF. *B* bronchus; *G* gastric tube

The patient was administered an antibiotic agent, and the right thoracic cavity was drained. Air did not leak, and turbid fluid did not flow from the thoracic tube. Bronchoscopy confirmed the presence of a fistula delivering digestive juice into the lung between the base of the right inferior lobe bronchus and the gastric tube (Fig. 3). Therefore, the severe pneumonia was considered to have occurred due to the reflux of digestive juice into the lung through the fistula. Furthermore, because air did not leak and turbid fluid did not flow from the thoracic tube, we considered that a rupture from the GBF into the pleural cavity had not occurred, and the pneumothorax on a CT was not secondary to the GBF, but was actually a mild spontaneous pneumothorax, because the patient developed a spontaneous pneumothorax several times previously due to severe emphysema.

The contents of the gastric tube were drained by inserting a nasogastric tube with suction. However, digestive juice frequently refluxed into the lung, and the respiratory failure persisted. We considered that conservative therapy alone would be insufficient to allow the patient to recover from such a terminal state, and thus, surgical intervention remained the most feasible option at 3 days after admission.

**Fig. 4:**
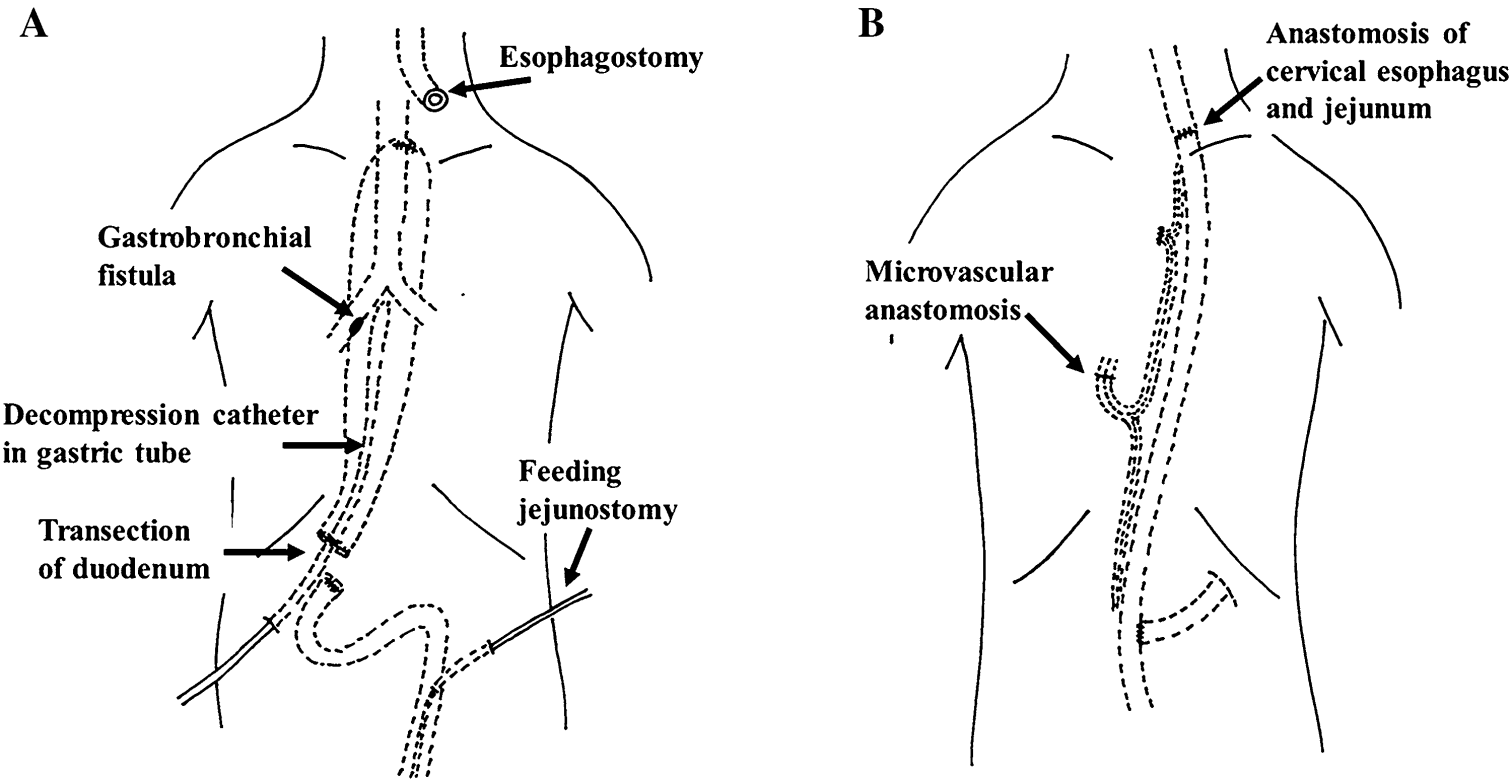
A schematic diagram of the two-stage operation. **a** The first procedure: the duodenum was transected, a decompression catheter was inserted into the gastric tube and esophagostomy established. **b** The second procedure: esophageal reconstruction was performed using a pedicled jejunum with a microvascular anastomosis through the subcutaneous route in the anterior chest wall

We considered that separating the gastric tube from the bronchus by repairing the defects would be very difficult in this patient, because the severe respiratory failure rendered an invasive procedure via a thoracotomy and one-lung ventilation impossible. Therefore, we decided to leave the GBF and separate the respiratory and digestive tracts without a thoracotomy. We transected the duodenum, inserted a decompression catheter into the gastric tube as a gastrostomy, brought the cervical esophagus to the left cervical region as an esophagostomy and closed the upper end of the gastric tube. A feeding jejunostomy was also constructed for postoperative nutritional management (Fig. 4a). At the end of the procedure, a tracheotomy was prepared, and artificial ventilation was managed with as low an airway pressure as possible.

This procedure stopped the reflux of digestive juice into the lung through the fistula, and the patient recovered from the severe pneumonia. The patient then underwent 2 months of management with antiulcer agents, physical rehabilitation and nutritional support to prepare him for reversing the esophageal discontinuity.

The esophageal defect was repaired via a secondary reconstruction 70 days after the first surgery. The esophagus was reconstructed using a pedicled jejunum with microvascular anastomosis in a Roux-en-Y fashion through a subcutaneous route in the anterior chest wall. The second and third branches of the jejunal vessels were divided and ligated near their roots, and the long segmented jejunum was subcutaneously pulled up together with a pedicle of the fourth branch. The internal thoracic artery and vein were then anastomosed to the third jejunal artery and vein, respectively, in the jejunal graft. The cervical esophagostomy was taken down, and the lifted ileum and cervical esophagus were manually anastomosed by hand sewing in the neck. The anal end of the pedicled jejunum was anastomosed to the jejunum in the Roux-en-Y configuration (Fig. 4b). The decompression tube was not removed at the secondary surgery, because it was considered necessary to remove gastric contents.

The patient started taking oral nutrition 20 days after the secondary reconstruction, and was discharged 38 days after the bypass.

## Discussion

The stomach is generally the first choice as an esophageal substitute after esophagectomy for patients with esophageal cancer. However, complications can be associated with using the gastric tube even in recurrence-free patients. Among such complications, the reported incidence of GBF ranges from 0.3 to 1.5 % [1–3].

The various reported causes of GBF are anastomotic leakage [3, 7], necrosis of the gastric tube [7], peptic ulcers [8], bronchial ischemia [9] and radiation [9]. We considered that the direct cause of the GBF in our case was the gastric tube ulcer in the present patient. Gastric tube ulcers have often been discovered by gastrointestinal fiberscopy after CRT for gastric tube cancer. Therefore, the occurrence of the ulcers in our patient was considered to be related to the CRT used to treat the gastric tube cancer. Because the gastric cancer was located behind the left atrium on CT, we thought the GBF occurred in a different site from the irradiated field used for the gastric tube cancer. Some patients have similarly developed gastric tube ulcer after CRT, [9, 10] indicating that radiation might produce direct mucosal injury or promote a reduction in mucosal blood flow to the gastric tube [11, 12].

Although the optimal reconstruction route for the gastric tube remains controversial, it has been reported that the posterior mediastinal route of reconstruction reduced the incidence of postoperative complications when compared with the retrosternal route [13]. However, several diseases of the gastric tube, such as cancer, ulcers and GBF, infrequently occur during long-term follow-up. Surgical resection for these diseases is difficult in patients with posterior mediastinal route reconstruction. From this point of view, the retrosternal route might be favorable for young patients who need long-term follow-up.

The site and size of the fistula, the underlying cause, and the clinical presentation influence how a GBF is managed. The approach can be conservative if the disease is mild [2, 3, 9], but early surgical treatment is frequently required even for patients without a severe status.

Surgical treatment generally consists of a direct approach with dissection of the fistula and closure of the tracheal and gastric tube defects [14, 15]. In addition, some authors have described using interposed vital tissue (diaphragm, pericardial or muscle-flaps) [1, 7, 8, 16–18] to fill dead space, protect the fistula between the bronchus and gastric tube and possibly to help prevent fistulae from recurring.

However, these definitive repairs of GBF require thoracotomy and one-lung ventilation, with a long surgical duration, and they are considered to be too invasive for patients with severe respiratory failure due to a GBF. Moreover, if the fistula fails to close after invasive surgery, the condition of the patient will steadily worsen. The surgery is also associated with high morbidity rates and potential mortality. Therefore, deciding whether or not to perform surgery in patients with a severe GBF is extremely difficult.

We considered that thoracotomy, one-lung ventilation and closure of the fistula with reinforcement by a myocutaneous flap in a previously irradiated field would be impossible in our patient because of the severe respiratory failure. Therefore, we selected the safer surgical procedure by laparotomy and the cervical procedure without thoracotomy. The patient recovered from the respiratory failure caused by the GBF, and then the second bypass operation restored the esophageal discontinuity. We selected a supercharged jejunal graft for reconstruction, and also use it as the first choice after esophagectomy when the stomach is not available in our institute.

## Conclusion

A GBF after esophagectomy is a serious and challenging complication. A two-stage operation resulted in a favorable outcome for our patient with GBF. This approach was safe and useful for this critically ill patient with respiratory failure due to a GBF. Appropriate, individual management according to the patient’s current status and the severity of the disease is important to determine the outcome.

## Abbreviations

CRT: Chemoradiation therapy
CT: Computed tomography
GBF: Gastro-bronchial fistula

## References

[1] Yasuda T, Sugimura K, Yamasaki M, Miyata H, Motoori M, Yano M (2012). Ten cases of gastro-tracheobronchial fistula: a serious complication after esophagectomy and reconstruction using posterior mediastinal gastric tube. Dis Esophagus.

[2] Bartels HE, Stein HJ, Siewert JR (1998). Tracheobronchial lesions following oesophagectomy: prevalence, predisposing factors and outcome. Br J Surg.

[3] Buskens CJ, Hulscher JB, Fockens P, Obertop H, van Lanschot JJ (2001). Benign tracheo-neo-esophageal fistulas after subtotal esophagectomy. Ann Thorac Surg.

[4] Aoki Y, Hihara J, Sakogawa K, Taomoto J, Hamai Y, Emi M (2012). Advanced esophageal cancer with an esophago-bronchial fistula successfully treated by chemoradiotherapy following esophageal bypass surgery: report of a case. Surg Today.

[5] Sobin LH, Wittekind C (2002). TNM classification of malignant tumours.

[6] Yoshida K, Ninomiya M, Takakura N, Hirabayashi N, Takiyama W, Sato Y (2006). Phase II study of docetaxel and S-1 combination therapy for advanced or recurrent gastric cancer. Clin Cancer Res.

[7] Nardella JE, Van Raemdonck D, Piessevaux H, Deprez P, Droissart R, Staudt JP (2009). Gastro-tracheal fistula—unusual and life threatening complication after esophagectomy for cancer: a case report. J Cardiothorac Surg.

[8] Kubota H, Hirai T, Matsumoto H, Murakami H, Higashida M, Hirabayashi Y (2009). A case of a gastrobronchial fistula after esophageal reconstruction successfully closed with an intercostal muscle flap. Esophagus.

[9] Martin-Smith JD, Larkin JO, O’Connell F, Ravi N, Reynolds JV (2009). Management of gastro-bronchial fistula complicating a subtotal esophagectomy: a case report. BMC Surg.

[10] Piessen G, Lamblin A, Triboulet JP, Mariette C (2007). Peptic ulcer of the gastric tube after esophagectomy for cancer: clinical implications. Dis Esophagus.

[11] McDermott M, Hourihane DO (1993). Fatal non-malignant ulceration in the gastric tube after oesophagectomy. J Clin Pathol.

[12] Aiko S, Ando N, Shinozawa Y, Ozawa S, Kitajima M, Kurose I (1993). Increased chemiluminescence and ulcer development in the low blood flow state of the gastric tube for esophageal replacement. J Clin Gastroenterol.

[13] Zheng YZ, Dai SQ, Li W, Cao X, Wang X, Fu JH (2012). Comparison between different reconstruction routes in esophageal squamous cell carcinoma. World J Gastroenterol.

[14] Pramesh CS, Sharma S, Saklani AP, Sanghvi BV (2001). Broncho-gastric fistula complicating transthoracic esophagectomy. Dis Esophagus.

[15] Fayoumi S, Sawalhi S (2007). Closure of tracheogastric fistula by video-assisted tracheoscopy, direct repair, and self-expandable titanium stent in a patient with total laryngopharyngoesophagectomy. J Thorac Cardiovasc Surg.

[16] Song SW, Lee HS, Kim MS, Lee JM, Kim JH, Zo JI (2006). Repair of gastrotracheal fistula with a pedicled pericardial flap after Ivor Lewis esophagogastrectomy for esophageal cancer. J Thorac Cardiovasc Surg.

[17] Kalmár K, Molnár TF, Morgan A, Horváth OP (2000). Non-malignant tracheo-gastric fistula following esophagectomy for cancer. Eur J Cardiothorac Surg.

[18] Jha PK, Deiraniya AK, Keeling-Roberts CS, Das SR (2003). Gastrobronchial fistula—a recent series. Interact Cardiovasc Thorac Surg.

